# Endophyte-mediated chromium detoxification in *Sorghum sudanense*: plant growth promotion and soil microbial enrichment

**DOI:** 10.3389/fmicb.2025.1734633

**Published:** 2026-01-21

**Authors:** Wenling Yang, Shujing Quan, Gao Lei, Ling Liu, Yongzhan Zhang, Jing Zhen, Qi Mu, Hongguang Xu, Huomiao Ran, Liangliang Li

**Affiliations:** 1 Institute of Biology Co., Ltd., Henan Academy of Sciences, Zhengzhou, Henan, China; 2 Key Laboratory of Microbial Engineering of Henan, Zhengzhou, Henan, China; 3 Langfang Yingcai School, Langfang, Hebei, China

**Keywords:** endophytic bacteria, heavy metal contamination, microbial community, phytoremediation, plant growth-promoting bacteria

## Abstract

Phytoremediation, assisted by endophytes, showed great promise for the efficient remediation of chromium (Cr)-contaminated soil. Three endophytic bacterial strains (SE16, SE19, and SE47) were isolated from various tissues of *Sorghum sudanense*. The pot experiment was designed to evaluate the potential of these endophytes, applied individually or in combination, to enhance the phytoremediation efficiency of *S. sudanense* in Cr-contaminated soil. The results demonstrated that inoculation with endophytes increased plant height and root length by 26.4–49.2% and by 63.5–122.8%, respectively. With the exception of the SE47 treatment, the fresh weights of the shoot increased significantly, reaching 2.01–3.08 times that of the non-inoculated control. Endophyte inoculation also led to a marked reduction in the Cr content in the shoots and roots of *S. sudanense*. The chlorophyll content increased, while the malonaldehyde (MDA) content decreased significantly after inoculation, indicating the alleviation of the cytotoxicity of Cr. The peroxidase (POD) activity in both the shoots and roots of *S. sudanense* decreased after inoculation. In shoots, catalase (CAT) activity was significantly lower in the combined inoculation treatments than in the non-inoculated control. In contrast, single inoculation treatments significantly increased CAT activity in roots compared to the control. Furthermore, endophyte inoculation increased soil organic matter (OM) and alkaline phosphatase activity. At the genus level, endophyte inoculation increased the relative abundance of *Delftia* and *Saccharimonadales*, which may contribute to reducing the toxic effects of heavy metals to plants. Our findings indicated that the endophytic bacteria are promising candidates for promoting plant growth and facilitating microbe-assisted phytoremediation in heavy metal-contaminated soil.

## 1 Introduction

As a leading global producer of chromium salts, the industry relies on chromium (Cr) and its compounds as essential raw materials in various sectors, including electroplating, leather tanning, textile dyeing and pigment production, metallurgy, and offset printing. Each year, substantial amounts of toxic Cr are released into the environment. In the natural environment, Cr primarily exists in two relatively stable forms: hexavalent Cr(VI) and trivalent Cr(III) (Ashraf et al., 2017). These two forms exhibit significant differences in terms of soil adsorption, bioavailability, plant uptake, and translocation, as well as phytotoxicity (Amin and Kassem, 2012; Choppala et al., 2018; Elzinga and Cirmo, 2010). Due to the low solubility of Cr(III) precipitates, only a small amount of toxic ions can pass through the cell membrane (Hu et al., 2021). In contrast, Cr(VI) is highly mobile, reactive, and toxic and is considered 1,000 times more noxious than Cr(III) (Hu et al., 2021). Owing to its non-degradability, solubility, mobility, and concealment, Cr(VI) can easily penetrate human cells, causing damage to internal organs such as the liver and kidneys, as well as to DNA (Malaviya and Singh, 2016; Mishra and Bharagava, 2016). Recognized internationally as a mutagenic and carcinogenic metal, Cr(VI) poses serious risks to both ecological systems and human health.

Phytoremediation, which employs plants to remove, stabilize, or detoxify heavy metals (HMs) *in situ*, has attracted increasing attention for its cost-effectiveness, operational simplicity, and environmental friendliness. Microorganisms can enhance plant remediation of HMs through mechanisms such as altering HM activity, promoting plant growth, and improving plant tolerance to HMs (Sahoo et al., 2024; Shahzad et al., 2019; Xu et al., 2016; Yung et al., 2021). Endophytes, which inhabit the rhizosphere or internal plant tissues, exhibit enhanced ability to coordinate population dynamics and adapt to both the environment and their host (He C. et al., 2020; Saharan et al., 2023). Notably, plant growth-promoting endophytes establish symbiotic associations with host plants that significantly strengthen phytoremediation capacity for HM contamination (Chen et al., 2010; Herpell et al., 2023; Kumar et al., 2021; Wang et al., 2022; Wang et al., 2023). These endophytes facilitate plant growth under HM stress by performing biological nitrogen (N) fixation, solubilizing phosphorus (P), and secreting phytohormones, organic acids, chelators, biosurfactants, siderophores, specific enzymes such as 1-aminocyclopropane-1-carboxylate (ACC) deaminase and chitinase, and antibiotics. Furthermore, they contribute to enhanced photosynthetic efficiency and increased plant biomass, thereby alleviating HM toxicity and improving plant resistance to HMs (Chi et al., 2024; Herpell et al., 2023; Jan et al., 2019; Liu et al., 2024; Schue et al., 2011; Wu et al., 2018).

Recent studies have shown that combined bioaugmentation inoculants composed of two or more plant growth-promoting bacteria (PGPB) were more conducive to plant growth and plant resistance to stress than single-strain PGPB inoculants (He C. et al., 2020; He X. et al., 2020; Jeyasundar et al., 2021; Ju et al., 2019; Liu et al., 2025; Rao et al., 2025). Some studies have shown that inoculation of root endophytic strains was more functional than rhizospheric strains and shoot endophytic strains (Pan et al., 2017; Wang Q. et al., 2020; Wang et al., 2019). It was reported that the original ecological niches were not a major factor in the growth-promoting attributes, Cd phytoextraction efficiency, and changes in bacterial community structure, suggesting that ecological niche was not the primary determinant for constructing effective bioaugmentation inoculants (Wang et al., 2022). Therefore, the principles for constructing efficient bioaugmentation inoculants have not yet been elucidated.

Recently, we isolated several endophytic bacterial strains with plant growth-promoting traits from *Sorghum sudanense*. *S. sudanense*, an annual forage grass of the genus Sorghum, is known for its high yield, superior quality, and strong adaptability. It exhibits rapid growth, high biomass production, robust stress tolerance, soil stabilization capacity, and efficient resource utilization. In recent years, *S. sudanense* has gained increasing attention as a promising plant for the phytoremediation of contaminated soils (Angelova et al., 2011). The objectives were (1) to investigate the synergistic effect of *S. sudanense* and its endogenous endophytes on the remediation of Cr-contaminated soil; (2) to evaluate the influence of single and combined inoculation of endophytes on phytoremediation efficiency from the response of both plant physiobiochemistry and soil microecology; and (3) to understand the mechanisms by which endophytic bacteria assist *S. sudanense* in remediating soil Cr contamination. The findings are expected to provide theoretical insights into the benefits of inoculating plants with beneficial microorganisms and the mutual adaptation mechanisms between endophytes and host plants.

## 2 Materials and methods

### 2.1 Materials

Three endophytic strains, SE16 (*Pantoea* sp., NCBI accession number PX642997), SE19 (*Priestia* sp., NCBI accession number PX642996), and SE47 (*Peribacillus* sp., NCBI accession number PX642995), with P-solubilizing, indoleacetic acid (IAA)--producing, and ACC deaminase-producing characteristics, were all screened from *S. sudanense* and stored in the Key Laboratory of Microbial Engineering of Henan (Henan, China). Previous antagonistic experiments showed that there were no antagonistic effects among the three strains. *S. sudanense* seeds were purchased from Zhengzhou Huiboyuan Horticulture Co., Ltd. (Henan, China).

### 2.2 Pot experiment design

Soil for the pot experiment was collected from the surface layer (0–20 cm) of a paddy field in Xinxiang, Henan province, China. The soil was air-dried, ground, and sieved through a 2-mm mesh. An aqueous solution of K₂Cr₂O₇ was prepared using autoclaved distilled water to achieve a contamination level of 300 mg Cr per kg of soil. The solution was applied to the soil and mixed thoroughly to ensure even distribution of the Cr. Subsequently, the soil was air-dried in shade and allowed to equilibrate for 2 months. One hundred and fifty *S. sudanense* seeds were sown in plastic pots containing 1 kg of Cr-contaminated soil. For endophytes inoculation, SE16, SE19, and SE47 suspensions were prepared by culturing these strains in Luria–Bertani (LB) liquid medium overnight at 37 °C to the exponential phase with continuous shaking. Following centrifugation to clear the medium, the endophyte inocula were prepared by suspending the cells in sterilized water to achieve an inoculum density of 10^9^ cfu/mL, respectively. Isochoric bacterial suspensions were prepared in a 1:1 or 1:1:1 ratio for inoculated treatments. Six treatment groups with three replicates were used. The specific design is shown in Table 1. For treatment groups inoculated with endophytes, the endophytes were gently inoculated into the Cr-contaminated soil before planting and after the emergence of *S. sudanense* with 50 mL of endophyte inoculum. For control without inoculation, 50 mL of sterile water was drenched. The pot experiment was conducted in an artificial climate chamber for 30 days under the following cultivation conditions: a photoperiod of a 16-h: 8-h light/dark cycle, a temperature of 25 ± 1 °C, and a relative humidity of 75%. Soil and plant samples were collected from each pot after 30 days of cultivation.

**Table 1:** The design of pot experiment.

Treatments	Cr concentration (mg/kg)	Endophyte inoculation	*S. sudanense*
CK	300	−	+
T1	300	SE16	+
T2	300	SE19	+
T3	300	SE47	+
T4	300	SE19 + 47	+
T5	300	SE16 + 19 + 47	+

“−” represent without inoculation (CK); “+” represent planting *S. sudanense*.

### 2.3 Soil analysis

#### 2.3.1 Soil physicochemical properties

Rhizosphere soil was collected for further DNA extraction according to Edwards et al. (2015). In addition, dried soil was used for the determination of physicochemical properties, Cr concentrations, and soil enzymatic activities.

The soil pH was determined at a 1:2.5 ratio of soil mass (2 g) to water volume (5 mL) using a pH meter (pH S-3C, Shanghai, China). Soil organic matter (OM) was determined by loss on ignition (550 °C, 4 h) (Schulte and Hopkins, 1996). Soil-hydrolyzed nitrogen was determined by the basic diffusion method (Bao, 2000). The Cr concentrations in soil were determined by an AA-6880F/AAC atomic absorption spectrophotometer (Shimadzu, Tokyo, Japan) with a wavelength of 283.3 nm. The activities of soil enzymes (urease, alkaline phosphatase, and CAT) were measured using detection kits from Beijing Solarbio Science and Technology Co., Ltd. (Beijing, China).

#### 2.3.2 Soil bacterial community

The microbial community genomic DNA was extracted from rhizosphere soil using the E.Z.N.A.^®^ soil DNA kit (Omega Bio-Tek Inc., Norcross, GA, United States) according to the manufacturer’s instructions. The DNA extract was checked on 1% agarose gel, and DNA concentration and purity were determined with a NanoDrop 2000 UV–Vis spectrophotometer (Thermo Scientific, Wilmington, United States). The hypervariable region V3–V4 of the bacterial 16S rRNA gene sequence was amplified with the universal primers 338F (5′-ACTCCTACGG-GAGGCAGCAG-3′) and 806R (5′-GGACTACHVGGGTWTCTAAT-3′) by an ABI GeneAmp^®^ 9,700 PCR thermocycler (ABI, CA, United States). The PCR amplification of the 16S rRNA gene was performed as follows: an initial denaturation at 95 °C for 3 min, followed by 27 cycles of denaturation at 95 °C for 30 s; annealing at 55 °C for 30 s; and extension at 72 °C for 45 s. A single final extension was carried out at 72 °C for 10 min, followed by termination at 4 °C. The PCR mixtures contain 4 μL of 5 × *TransStart* FastPfu buffer, 2 μL of 2.5 mM dNTPs, 0.8 μL of forward primer (5 μM), 0.8 μL of reverse primer (5 μM), 0.4 μL of *TransStart* FastPfu DNA Polymerase, 10 ng of template DNA, and finally, ddH_2_O was diluted to 20 μL. PCR reactions were performed in triplicate. The PCR product was extracted from 2% agarose gel, purified using the AxyPrep DNA Gel Extraction Kit (Axygen Biosciences, Union City, CA, United States), according to the manufacturer’s instructions, and quantified using Quantus™ Fluorometer (Promega, United States).

Purified amplicons were pooled in equimolar and paired-end sequenced on an Illumina MiSeq PE300 platform/NovaSeq PE250 platform (Illumina, San Diego, United States) according to the standard protocols by Majorbio Bio-Pharm Technology Co. Ltd. (Shanghai, China).

The raw 16S rRNA gene sequencing reads were demultiplexed, quality-filtered by fastp version 0.20.0 (Chen et al., 2018), and merged by FLASH version 1.2.7 (Magoč and Salzberg, 2011) with the following criteria: (i) the 300 bp reads were truncated at any site receiving an average quality score of < 20 over a 50-bp sliding window, and the truncated reads shorter than 50 bp were discarded, reads containing ambiguous characters were also discarded; (ii) only overlapping sequences longer than 10 bp were assembled according to their overlapped sequence. The maximum mismatch ratio of the overlap region is 0.2. Reads that could not be assembled were discarded; and (iii) samples were distinguished according to the barcode and primers, and the sequence direction was adjusted using exact barcode matching, allowing up 2 nucleotide mismatch in primer matching.

Operational taxonomic units (OTUs) with 97% similarity cutoff (Edgar, 2013; Stackebrandt and Goebel, 1994) were clustered using UPARSE version 7.1 (Edgar, 2013), and chimeric sequences were identified and removed. The taxonomy of each OTU representative sequence was analyzed by RDP Classifier version 2.2 (Wang et al., 2007) against the 16S rRNA database using a confidence threshold of 0.7.

### 2.4 Plant analysis

Each plant sample was subdivided into shoots and roots. The shoots and roots were separated, washed with deionized water, and weighed to measure their fresh weight. They were then heat-killed at 105 °C for 30 min and oven-dried at 75 °C for 48 h to estimate Cr accumulation in the plant. Part of the fresh shoots and roots were taken, washed with distilled water, immediately frozen in liquid nitrogen, and stored at −80 °C for the analysis of chlorophyll content, malondialdehyde (MDA) content, peroxidase (POD), and catalase (CAT) activity.

The chlorophyll content of the shoot was determined following the method of Lichtenthaler (1987). The MDA content was determined by homogenizing samples in 10% TCA, followed by centrifugation at 10,000 *g* for 10 min. Thiobarbituric acid (0.6%) and supernatant were mixed and then heated at 100 °C for 15 min, followed by cooling and centrifugation at 10,000 *g* for 15 min. The supernatant absorbance was recorded at 450, 532, and 660 nm (Chen et al., 2017). The activities of POD and CAT were measured using kits provided by Nanjing Jiancheng Bioengineering Institute, China. The Cr content of plants was determined by nitric acid–perchloric acid digestion–flame photometry.

The bioconcentration factor (BCF) and translocation factor (TF) were calculated according to the following Equations 1, 2:


BCF=Crconcentration in plants/Crconcentration in soils (1)

$$\begin{array}{l} \mathrm{TF}=\mathrm{Cr}\;\text{concentration in aboveground parts}/\\ \mathrm{Cr}\;\text{concentration in roots} \end{array}$$ (2)

### 2.5 Data analysis

Data are presented as the mean ± standard deviation (mean ± SD). Statistical analyses were performed using a one-way analysis of variance (ANOVA) followed by least significant difference (LSD) tests with SPSS software. Differences were considered significant at a *p*-value of ≤ 0.05.

## 3 Results

### 3.1 Effects of endophytes on plant growth and Cr accumulation

**Figure 1:**
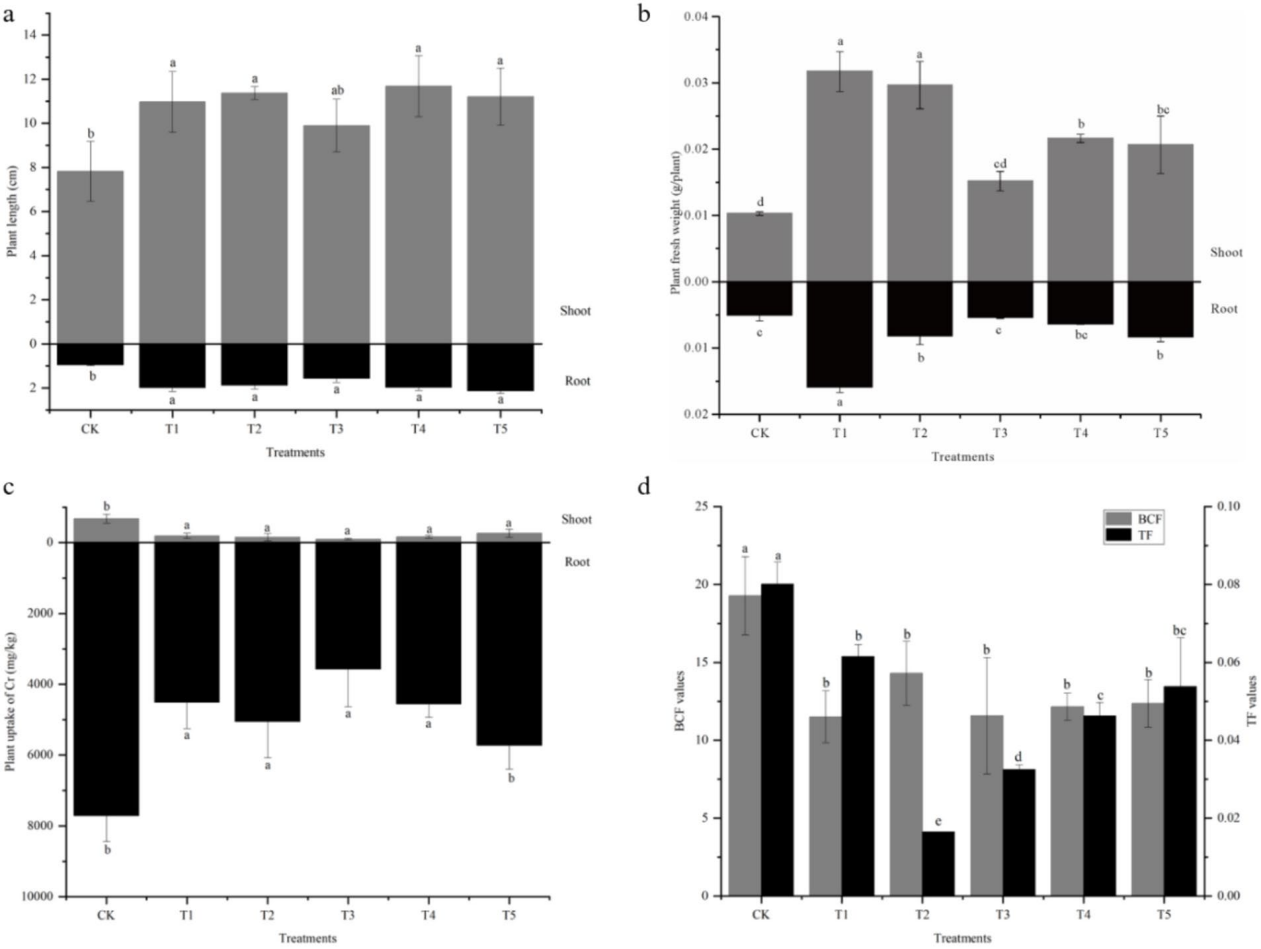
Effects of endophyte inoculation on the growth and Cr accumulation in *S. sudanense* under Cr stress (**a**: Length of shoot and root; **b**: Fresh weight; **c**: Cr accumulation; **d**: BCF and TF). Different letters indicate significant differences between the treatments (*p* < 0.05).

Pot experiments demonstrated that inoculation with endophytes under Cr stress significantly promoted the growth of *S. sudanense* (Figure 1). Both single and combined inoculations significantly increased shoot length (plant height) and root length. Compared with the non-inoculated control, plant height increased by 26.4–49.2%, and root length increased by 63.5–122.8% after inoculation with endophytes. Except for treatment T3, shoot fresh weights increased significantly, reaching 2.01–3.08 times that of the non-inoculated control. Root fresh weights were also significantly increased by inoculation, except in treatments T3 and T4.

The Cr uptake by *S. sudanense* grown in artificially contaminated conditions is shown in Figure 1. With or without inoculation of endophytes, the roots exhibited a higher Cr concentration compared to the shoots. Endophyte inoculation led to a marked reduction of Cr concentration in the shoots and roots of *S. sudanense*. Specifically, shoot Cr concentration was 2.59 to 6.93 times higher in non-inoculated control plants than in inoculated plants, while root Cr concentration in most inoculated treatments (except for the combined inoculation T5) decreased by 25.8 to 53.7% relative to the control. Furthermore, both the BCF and TF for Cr significantly decreased in all inoculation treatments compared with the non-inoculated control.

### 3.2 Effects of endophytes on plant physiology and biochemistry under Cr stress

#### 3.2.1 Photosynthesis

**Figure 2:**
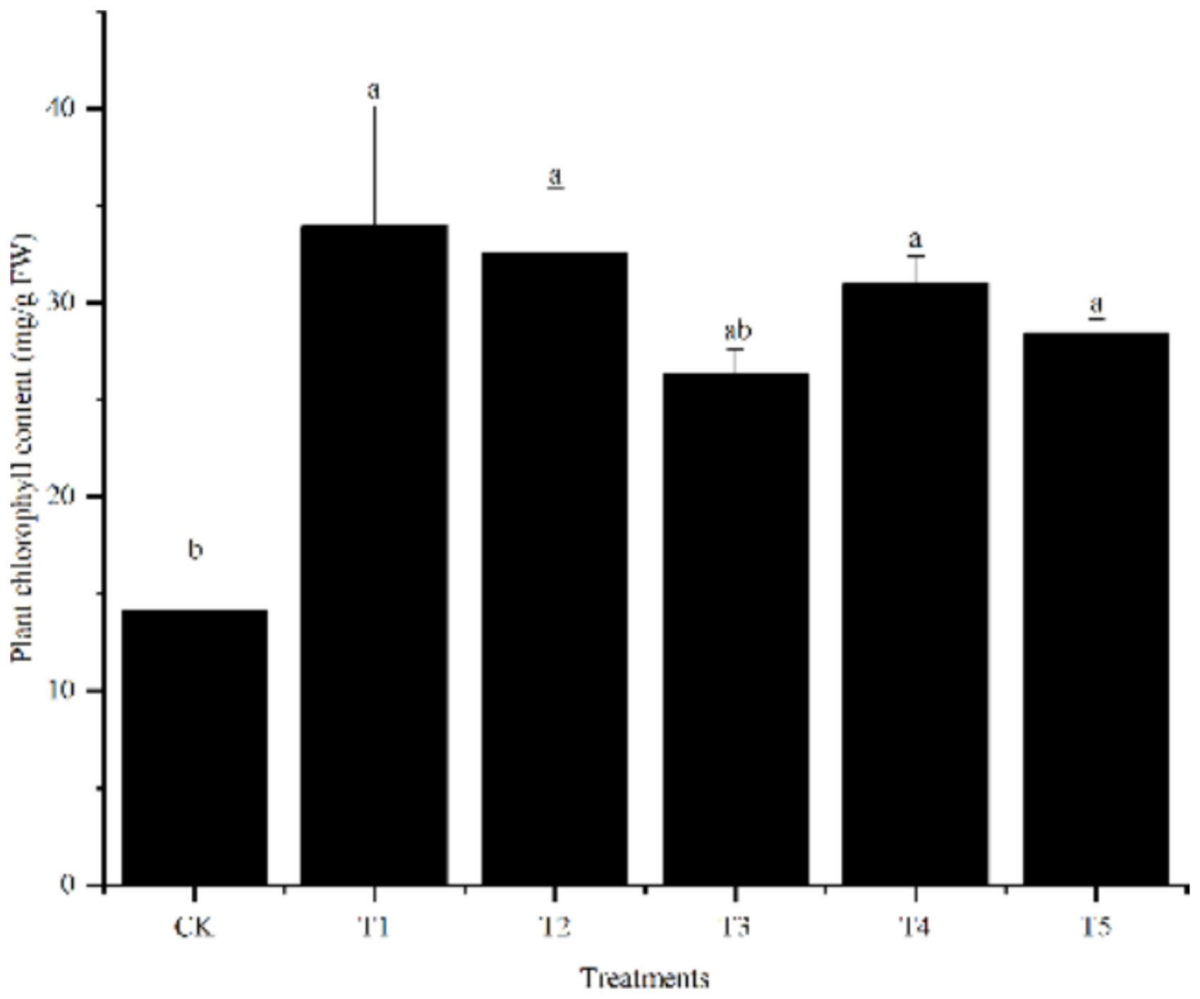
Effects of endophyte inoculation on the chlorophyll content in shoots of *S. sudanense* under Cr stress. Different letters indicate significant differences between the treatments (*p* < 0.05).

To investigate the effect of endophytes on photosynthesis in *S. sudanense* under Cr stress, the chlorophyll content in shoots was measured under different inoculation treatments (Figure 2). The results showed that, with the exception of T3, all other inoculation treatments significantly increased the chlorophyll content compared to the non-inoculated control, reaching levels 2.01–2.41 times that of the control.

#### 3.2.2 Antioxidant system

**Figure 3:**
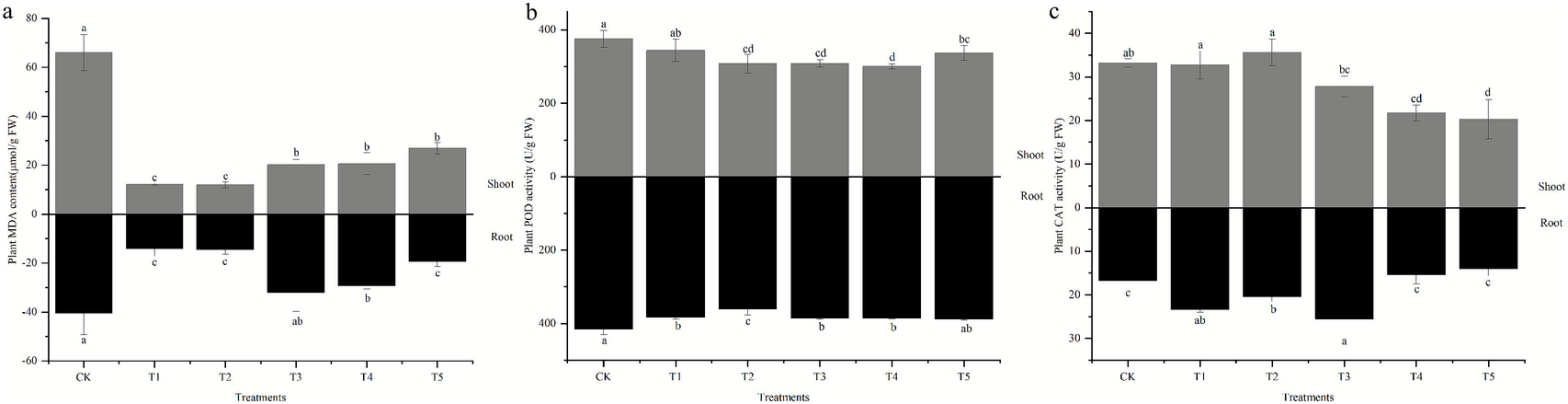
Effects of endophyte inoculation on MDA content **(a)**, and the activities of POD **(b)** and CAT **(c)** in shoots of *S. sudanense* under Cr stress. Different letters indicate significant differences between the treatments (*p* < 0.05).

As shown in Figure 3, endophyte inoculation reduced the MDA content in the plants. After inoculation, the MDA content in the shoots of *S. sudanense* was significantly lower than that in the non-inoculated control, particularly in the single inoculation treatments T1 and T2. With the exception of T3, the MDA content in the roots of all other inoculation treatments was also significantly reduced compared to the non-inoculated control.

Additionally, POD activity in both the shoots and roots of *S. sudanense* decreased after inoculation. All inoculation treatments, except for T1, resulted in a reduction of shoot POD activity by 10.27–20.05%. The POD activity in the roots decreased by 7.25–13.28% in all treatments, except for the combined inoculation T5.

Furthermore, CAT activity in the shoots of *S. sudanense* was significantly lower in the combined inoculation treatments compared to the non-inoculated control. In contrast, single inoculation treatments led to a significant increase in root CAT activity relative to the non-inoculated control.

### 3.3 Effects of endophytes on soil properties under Cr stress

#### 3.3.1 Soil physicochemical properties and Cr speciation

**Figure 4:**
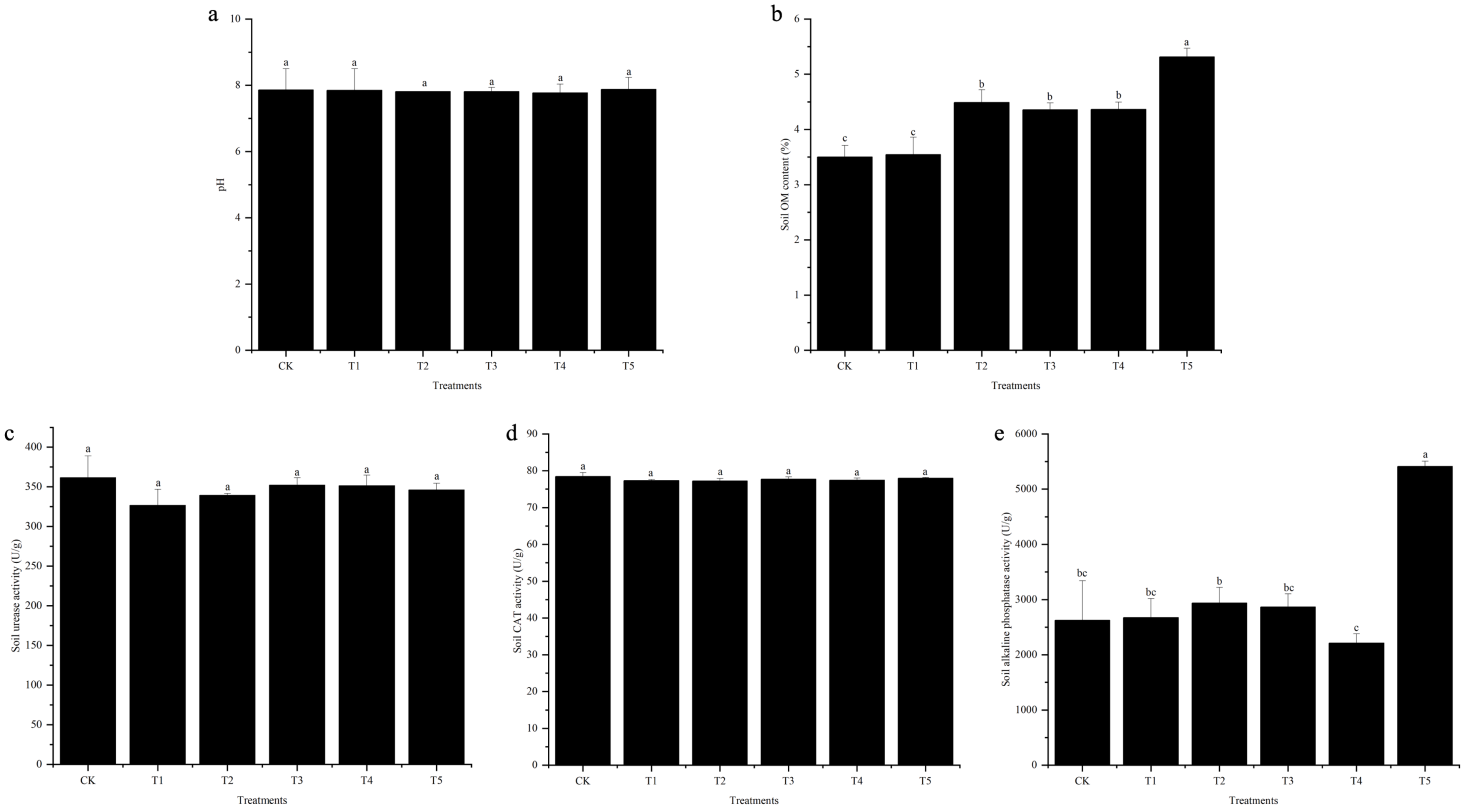
Effects of endophyte inoculation on soil properties under Cr stress (**a**: pH, **b**: OM, **c**: Urease activity, **d**: Catalase activity, **e**: Alkaline phosphatase activity). Different letters indicate significant differences between the treatments (*p* < 0.05).

The influence of endophyte inoculation on soil physicochemical properties was investigated, and the results are shown in Figure 4. Under Cr stress, the influence of endophyte inoculation on soil pH showed no significant differences. Inoculation with endophytes increased the soil OM under Cr stress. Except for the T1 treatment, the differences in soil OM between all other inoculation treatments and the non-inoculated control reached a significant level (*p* < 0.05). In particular, the combined inoculation treatment T5 increased OM by 51.86% compared to the non-inoculated control. Under Cr stress, the effects of endophyte inoculation on soil CAT and urease activities were relatively minor. However, the activity of soil alkaline phosphatase in the combined inoculation treatment T5 significantly increased compared to the non-inoculated control, with the difference reaching a significant level (*p* < 0.05).

**Figure 5:**
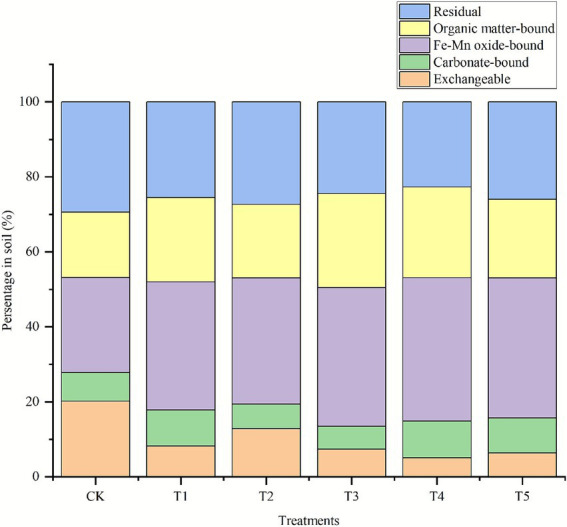
Effects of endophyte inoculation on Cr speciation in soil.

The Cr speciation of rhizosphere soil was detected, and the result is shown in Figure 5. After endophyte inoculation, the proportion of exchangeable Cr decreased, while the proportion of Fe-Mn oxide-bound Cr and organic matter-bound Cr increased compared with the non-inoculated control. The proportion of carbonate-bound Cr in soil treated by single inoculation T1 and combined inoculation increased.

#### 3.3.2 Analysis of soil bacterial community

High-throughput sequencing technology was employed to investigate the microbial community in soils under different treatment groups. The alpha-diversity index of soil bacteria under Cr stress was evaluated after inoculation with endophytes (Table 2). Compared with the non-inoculated control, inoculation with endophytes, except for T2, induced a decline of the Shannon, Ace, and Chao indices, and an upward trend of the Simpson index. While the inoculation with endophytes had no significant effect on Shannon and Simpson indices of the bacterial community in soil, the combined inoculation T5 decreased the Ace index by 19.79% and the Chao index by 20.88% (Table 1), indicating that the combined inoculation T5 did not change the community diversity but could alter the bacterial richness in soil.

**Table 2:** Effects of endophyte inoculation on *α*-diversity of bacterial community in soil under Cr stress.

Treatment	Shannon	Simpson	Ace	Chao
CK	4.27 ± 0.41ab	0.4327 ± 0.01a	1508.59 ± 81.27b	1568.89 ± 162.54ab
T1	4.03 ± 0.37ab	0.9624 ± 0.05a	1470.74 ± 7.25c	1456.70 ± 138.33abc
T2	4.62 ± 0.15a	0.3521 ± 0.01a	1725.75 ± 26.50a	1652.39 ± 92.49a
T3	3.83 ± 0.60b	0.9399 ± 0.04a	1435.02 ± 134.67bc	1288.20 ± 181.16c
T4	4.00 ± 0.45ab	0.7221 ± 0.02a	1420.29 ± 53.23c	1370.98 ± 23.50bc
T5	3.81 ± 0.18b	0.6813 ± 0.02a	1210.02 ± 93.42 cd	1241.27 ± 184.64c

Data are means ± SD (*n* = 3). Different letters denote significant differences between the treatments (*p* < 0.05).

**Figure 6:**
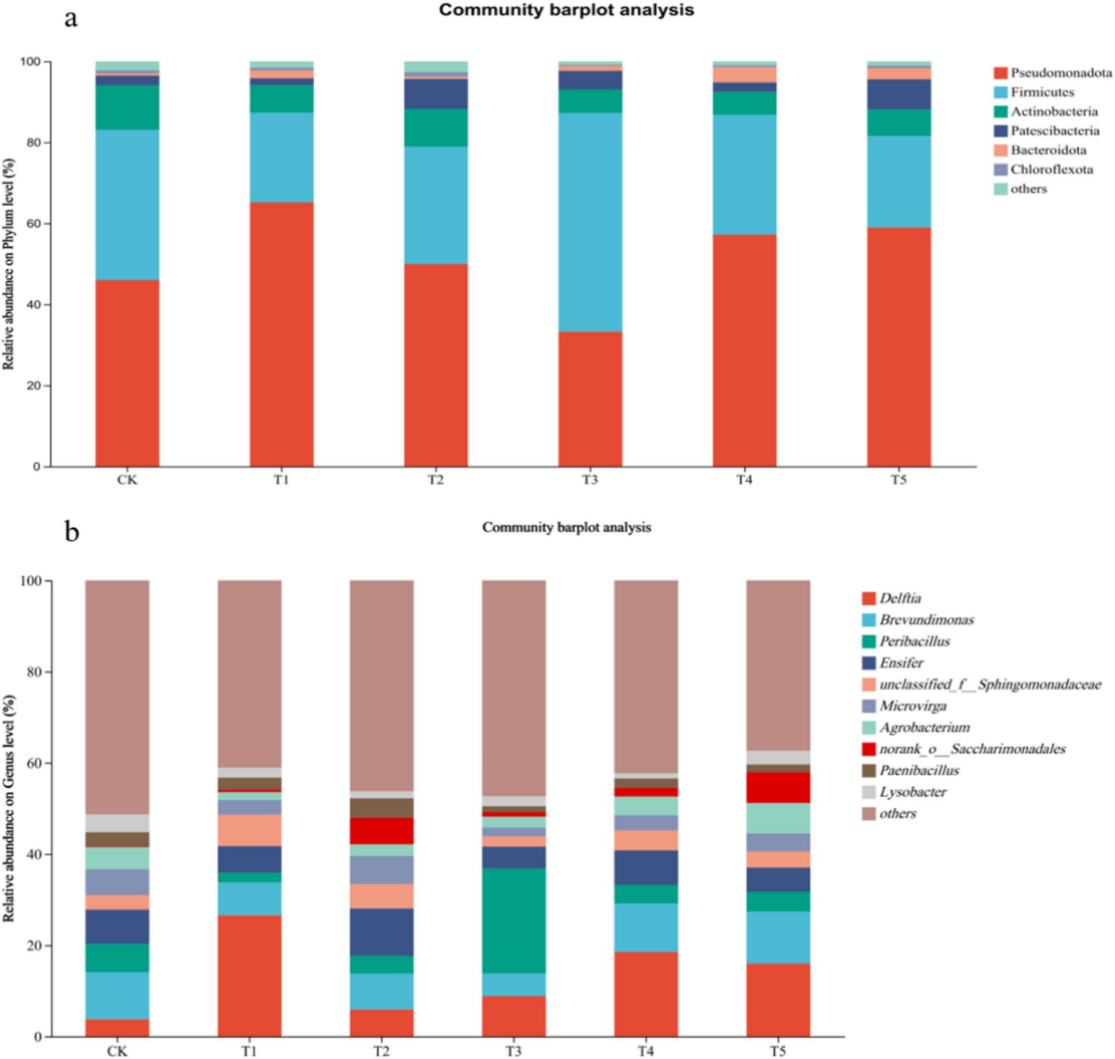
Effects of endophyte inoculation on bacterial community composition in soil under Cr stress (**a**: Phylum-level species composition; **b**: Genus-level species composition).

High-throughput sequencing results of *S. sudanense* rhizosphere soil samples were analyzed. As shown in Figure 6, under Cr stress, the dominant genera in the rhizosphere soil of *S. sudanense* across different treatments were *Delftia*, *Brevundimonas*, *Peribacillus*, *Ensifer*, *Microvirga*, *Agrobacterium*, *Saccharimonadales*, *Paenibacillus*, *Lysobacter*, and *Clostridium*.

The relative abundance analysis of soil bacterial phylum level showed that endophyte inoculation did not change the predominant bacterial species in the soil under Cr stress, but it did result in significant changes in their relative abundance. The relative abundance of Pseudomonadota and Bacteroidota in soil treated by single inoculation T1 and combined inoculation increased significantly compared to the non-inoculated control treatment.

At the soil bacterial genus level, it is worth noting that the relative abundance of *Delftia* was 3.93% in the soil of the non-inoculated control treatment, while it ranged from 6.19 to 27.46% in the soils of endophyte inoculation treatments. The relative abundance of *Saccharimonadales* was 0.14% in the soil of the non-inoculated control treatment compared to 0.56 to 6.77% in the soils of inoculation treatments. This finding indicated that the inoculation of endophytic bacteria promoted the enrichment of *Delftia* and *Saccharimonadales*.

## 4 Discussion

### 4.1 Effect of endophyte on soil properties

As key indicators of soil fertility and remediation status, soil enzymes are strongly influenced by pH, OM, and microbial activity. Consistent with previous findings, endophyte inoculation promoted soil microbial activity and further improved soil enzyme activity and fertility (Babu et al., 2015; Liu et al., 2022; Wang et al., 2022). In our study, although soil pH remained unaffected across treatments, inoculation increased the soil OM under Cr stress. Moreover, the combined inoculation treatment T5 resulted in a significant increase in alkaline phosphatase activity relative to the non-inoculated control.

The bioavailability of HMs in soil, which in turn determines phytoextraction efficiency, is primarily governed by their chemical speciation. In this study, endophyte inoculation was found to reduce the fraction of exchangeable Cr, thereby decreasing its bioavailability. This shift in speciation implied that the endophytes facilitated the stabilization of Cr in the soil.

### 4.2 Effect of endophyte on soil bacterial community

The composition of the soil microbial community is a key factor influencing the toxicity of HMs and plant tolerance during the remediation process (Zhang et al., 2015). Given this factor, soil microbial community structure is increasingly used as an indicator to assess the effectiveness of phytoremediation in contaminated soils (Gao et al., 2025; Li et al., 2022; Lin et al., 2025). Under HM stress, plant growth is strongly dependent on soil microorganisms, which facilitate nutrient acquisition and enhance metal tolerance through various growth-promoting mechanisms (Babu et al., 2013; Bacon and White, 2016; Chen et al., 2020; Yung et al., 2021).

Alpha diversity indices, such as the Shannon, Simpson, Ace, and Chao indices, are widely used to assess the diversity and richness of a microbial community. Previous studies have demonstrated that inoculation with endophytes (either bacterial or fungal, alone or in combination) under HM stress typically enhanced phytoremediation efficiency by increasing the diversity and richness of the soil microbial community (Gao et al., 2025; Wang L. et al., 2020; Wang et al., 2022). While our findings revealed that the endophyte inoculation led to a reduction in both soil bacterial diversity and richness, this discrepancy may be attributed to the competitive advantage of the introduced endophytes, which could have occupied ecological niches and consumed resources, thereby suppressing native microorganisms. Additionally, the inoculation might have intensified host selective pressure, further shaping the microbial community structure (Jiang R. et al., 2025; Lin et al., 2025). Plant root-driven changes in rhizosphere microbial communities have been well-documented (Bulgarelli et al., 2012; Edwards et al., 2015). The reduction in bacterial community diversity in the rhizosphere soil of certain plant systems has been suggested to result from a combined decline in both taxonomic richness and evenness (Li et al., 2012; Shi et al., 2015). Soil pH, the speciation of heavy metals, and certain physicochemical properties such as N and potassium (K) were major factors influencing the microbial community structure in rhizosphere soil (Wang et al., 2022). Our study indicated that inoculating endophytes altered the physicochemical properties of *S. sudanense* rhizosphere soil and changed the speciation of chromium, which inevitably modified the root environment of *S. sudanense*. Hou et al. (2018) suggested that plant roots may selectively influence surrounding microorganisms, altering species abundance and reducing the evenness of taxonomic distribution (enriching specific taxa or causing the disappearance of low-abundance taxa), thereby leading to a decline in taxon-based diversity indices. Our findings also confirmed this point. Although the inoculation of endophytes reduced soil microbial diversity and abundance, it promoted the enrichment of microorganisms associated with plant heavy metal resistance. Additionally, bacterial growth rate and biomass production are simultaneously influenced by metal ion concentrations (Deng et al., 2007; Hou et al., 2018). Certain sensitive taxa may be diminished due to heavy metal toxicity (Pishchik et al., 2016), which is also one of the reasons for the reduction in diversity.

The relative abundance analysis of soil bacterial phylum level showed that endophyte inoculation resulted in significant changes in their relative abundance, especially Pseudomonadota and Bacteroidota, in soil treated by single inoculation T1 and combined inoculation. Pseudomonadota, a highly diverse and abundant phylum, exhibited strong potential in the detoxification of Mn(II), Hg(II), As(III), and Cr(VI) into their less harmful forms (Belzile et al., 2006; Biełło et al., 2023; Campos et al., 2010; He et al., 2023; Naziębło and Dobrzyński, 2025; Yao et al., 2020). Bacteroidota possessed genes related to HM resistance, such as those encoding metal transport proteins, antioxidant enzymes, and other protective mechanisms, and could utilize various organic and inorganic substances as energy sources and enable themselves to survive in stressful environments (Jiang R. et al., 2025). Together, these microbes contribute to the alleviation of HM toxicity and improvement of environmental quality.

Endophyte inoculation significantly led to the enrichment of some bacterial genera, such as *Delftia* and *Saccharimonadales*. The genera *Delftia* and *Saccharimonadales* are known to play a significant role in the bioremediation of pollutants. They can alleviate HM stress on plants through multiple mechanisms, such as secreting phytohormones like IAA, transforming HMs into less toxic forms, and releasing specific compounds to enhance plant nutrient uptake (Adarme-Duran et al., 2025; Chandwani et al., 2025; Conlon et al., 2025; Doolotkeldieva et al., 2024; Long et al., 2024; Lyu et al., 2021; Zheng et al., 2024). These findings suggest that the inoculation of endophytic bacteria promoted the enrichment of *Delftia* and *Saccharimonadales*, which in turn support the growth of the plants and enhance the resistance of plants to Cr.

### 4.3 Effect of endophyte on plant growth

Endophytes enhance the phytoremediation efficiency by altering the HM activity, mitigating their toxicity and metal-induced stress on plants, promoting plant growth, and improving the resistance of plants to HMs (Badawy et al., 2022; Gao et al., 2025; Herpell et al., 2023; Lin et al., 2025; Liu et al., 2024; Ren et al., 2025; Wang et al., 2022; Ahsan et al., 2019; Xiang et al., 2024). Our results demonstrated that the endophyte inoculation increased the plant biomass, reduced the Cr concentration in the shoot and root of *S. sudanense*, enhanced plant photosynthetic capacity, and alleviated HM-induced oxidative stress, thereby promoting phytoremediation efficiency.

Our results showed that, although *Peribacillus* sp. SE47 possesses growth-promoting traits such as P solubilization, IAA production, and ACC deaminase activity, it did not enhance the biomass of *S. sudanense* in pot experiments. *Peribacillus frigoritolerans* was proven to stimulate plant growth under sterile conditions, but it failed to exhibit such effects in non-sterile environments (Świątczak et al., 2024). This may be due to the native rhizosphere microbial communities’ presence, resulting in the variation of beneficial effects of PGPB on plant development (Pacheco da Silva et al., 2022). Furthermore, the growth-promoting effect of the combined microbial community SE19 + 47 did not improve significantly, likely because complex interactions occurred among the strains after the consortium was constructed (Jiang W. et al., 2025), leading to the expression changes of functional genes in strain SE19, such as those involved in plant growth promotion, under the control of regulatory networks (Geesink et al., 2024).

Heavy metal stress will also lead to lipid peroxidation and potential depolarization of the cytoplasmic membrane, thereby affecting the composition, structure, and permeability of the cell membrane, resulting in intracellular electrolyte leakage (Ullah et al., 2019). MDA, the product of lipid peroxidation, serves as a key indicator of oxidative stress (Babu et al., 2015). In this study, MDA contents both in the shoot and root of *S. sudanense inoculated with* endophytes decreased under Cr stress. Heavy metal exposure induces the production of reactive oxygen species (ROS), resulting in oxidative damage to the plant, such as lipid peroxidation. To mitigate such damage, plants activate antioxidant defense systems, in which both antioxidant enzymes and non-enzymatic antioxidants play crucial roles (Ullah et al., 2019). While numerous studies have declared that endophytic bacteria can stimulate the synthesis of antioxidant enzymes in plants to alleviate oxidative stress (Bilal et al., 2019; Shahzad et al., 2019; Wang et al., 2023; Yue et al., 2021), our findings revealed a decrease in the activities of POD and CAT. This discrepancy may be attributed to the fact that endophyte-induced increases in plant biomass diluted the internal Cr concentration, thereby reducing HM toxicity and the corresponding demand for enzymatic detoxification (Khan et al., 2015). Furthermore, endophytic bacteria can mitigate the phytotoxicity of HMs, alleviating HM-induced oxidative stress and antioxidant responses in plants (Ma et al., 2016; Ullah et al., 2019; Wang et al., 2023).

Endophytic bacteria could promote the accumulation of HMs and root–shoot transfer in plants. However, the transfer would be influenced by factors such as HM bioavailability, physicochemical properties, soil HM composition, as well as the species of both the plant and the endophytic bacteria (Babu et al., 2015; Fan et al., 2018; Ma et al., 2011; Ma et al., 2016; Wang et al., 2023). In this study, endophyte inoculation significantly reduced Cr concentration in the shoots and roots of *S. sudanense*, with roots consistently showing higher Cr concentration than shoots. Plants with BCF > 1 and TF < 1 can be applied for phytostabilization (Babu et al., 2015), so our results demonstrated the potential of *S. sudanense* for Cr stabilization in plant roots.

## 5 Conclusion

The endophytic bacteria (SE16, SE19, and SE47) from *S. sudanense* exhibited good plant growth-promoting traits. Pot experiments demonstrated that *S. sudanense* primarily functioned to retain Cr in roots when inoculated with these endophytes, either individually or in combination. Endophyte inoculation increased plant height, root length, and fresh weight; improved photosynthetic performance; and alleviated HM-induced oxidative stress under Cr stress. Although a reduction in soil bacterial diversity and richness was observed after inoculation, the treatment promoted the enrichment of key beneficial genera, such as *Delftia* and *Saccharimonadales*, which contributed to plant growth and enhanced Cr resistance. These findings indicated that the endophytic bacteria are promising candidates for enhancing plant growth and facilitating microbe-assisted phytoremediation in HM-contaminated soil. These beneficial microorganisms can influence the soil microenvironment, play a role in soil biogeochemical cycles and ecological processes, and promote plant growth and crop yields, while also having a beneficial and lasting impact on sustainable agriculture.

## Data Availability

The data presented in this study are publicly available. The data can be found here: https://www.ncbi.nlm.nih.gov, accession numbers PX642997, PX642996 and PX642995.

## References

[ref1] Adarme-Duran C. A. Castillo E. Brandão P. F. B. (2025). Cadmium removal and indole acetic acid production by ureolytic bacteria isolated from rhizosphere soils. World J. Microbiol. Biotechnol. 41:302. doi: 10.1007/s11274-025-04482-9, 40779092 PMC12334442

[ref2] Ahsan M. T. Tahseen R. Ashraf A. Mahmood A. Najam-Ul-Haq M. Arslan M. . (2019). Effective plant-endophyte interplay can improve the cadmium hyperaccumulation in *Brachiaria mutica*. World J. Microbiol. Biotechnol. 35:188. doi: 10.1007/s11274-019-2757-z 31741120, 31741120

[ref3] Amin A. S. Kassem M. A. (2012). Chromium speciation in environmental samples using a solid phase spectrophotometric method. Spectrochim. Acta A Mol. Biomol. Spectrosc. 96, 541–547. doi: 10.1016/j.saa.2012.05.020, 22766579

[ref4] Angelova V. Ivanova R. Delibaltova V. Ivanov K. (2011). Use of Sorghum crops for *in situ* phytoremediation of polluted soils. J. Agric. Sci. Technol. A 1, 693–702.

[ref5] Ashraf A. Bibi I. Niazi N. K. Ok Y. S. Murtaza G. Shahid M. . (2017). Chromium(VI) sorption efficiency of acid-activated banana peel over organo-montmorillonite in aqueous solutions. Int. J. Phytoremediation 19, 605–613. doi: 10.1080/15226514.2016.1256372, 27849143

[ref6] Babu A. G. Kim J.-D. Oh B.-T. (2013). Enhancement of heavy metal phytoremediation by *Alnus firma* with endophytic *Bacillus thuringiensis* GDB-1. J. Hazard. Mater. 250-251, 477–483. doi: 10.1016/j.jhazmat.2013.02.014, 23500429

[ref7] Babu A. G. Shea P. J. Sudhakar D. Jung I.-B. Oh B.-T. (2015). Potential use of *Pseudomonas koreensis* AGB-1 in association with *Miscanthus sinensis* to remediate heavy metal (loid)-contaminated mining site soil. J. Environ. Manag. 151, 160–166. doi: 10.1016/j.jenvman.2014.12.045, 25575343

[ref8] Bacon C. W. White J. F. (2016). Functions, mechanisms and regulation of endophytic and epiphytic microbial communities of plants. Symbiosis 68, 87–98. doi: 10.1007/s13199-015-0350-2

[ref9] Badawy I. H. Hmed A. A. Sofy M. R. Al-Mokadem A. Z. (2022). Alleviation of cadmium and nickel toxicity and phyto-stimulation of tomato plant L. by endophytic *Micrococcus luteus* and *Enterobacter cloacae*. Plants 11:2018. doi: 10.3390/plants11152018, 35956496 PMC9370581

[ref10] Bao S. D. (2000). Agriculture and chemistry analysis of soil. Beijing: China Agricultural Press.

[ref11] Belzile N. Wu G. J. Chen Y.-W. Appanna V. D. (2006). Detoxification of selenite and mercury by reduction and mutual protection in the assimilation of both elements by *Pseudomonas fluorescens*. Sci. Total Environ. 367, 704–714. doi: 10.1016/j.scitotenv.2006.03.008, 16626785

[ref12] Biełło K. A. Olaya-Abril A. Cabello P. Rodríguez-Caballero G. Sáez L. P. Moreno-Vivián C. . (2023). Quantitative proteomic analysis of cyanide and mercury detoxification by *Pseudomonas pseudoalcaligenes* CECT 5344. Microbiol. Spectr. 11:e0055323. doi: 10.1128/spectrum.00553-23, 37432117 PMC10433974

[ref13] Bilal S. Shahzad R. Khan A. L. Al-Harrasi A. Kim C. K. Lee I.-J. (2019). Phytohormones enabled endophytic *Penicillium funiculosum* LHL06 protects *Glycine max* L. from synergistic toxicity of heavy metals by hormonal and stress-responsive proteins modulation. J. Hazard. Mater. 379:120824. doi: 10.1016/j.jhazmat.2019.120824, 31271935

[ref14] Bulgarelli D. Rott M. Schlaeppi K. Ver Loren Van Themaat E. Ahmadinejad N. Assenza F. . (2012). Revealing structure and assembly cues for *Arabidopsis* root-inhabiting bacterial microbiota. Nature 488, 91–95. doi: 10.1038/nature11336, 22859207

[ref15] Campos V. L. Valenzuela C. Yarza P. Kämpfer P. Vidal R. Zaror C. . (2010). *Pseudomonas arsenicoxydans* sp nov., an arsenite-oxidizing strain isolated from the Atacama desert. Syst. Appl. Microbiol. 33, 193–197. doi: 10.1016/j.syapm.2010.02.007, 20409659

[ref16] Chandwani S. Ahire V. Manoharadas S. Amaresan N. (2025). Cobalt tolerant bacteria mobilize iron in garden pea (*Pisum sativum* L.) to mitigate cobalt stress in iron deficit soils. Int. J. Phytoremediation 27:1833. doi: 10.1080/15226514.2025.2522304, 40556261

[ref17] Chen Q.-L. Ding J. Zhu Y.-G. He J.-Z. Hu H.-W. (2020). Soil bacterial taxonomic diversity is critical to maintaining the plant productivity. Environ. Int. 140:105766. doi: 10.1016/j.envint.2020.105766, 32371308

[ref18] Chen L. Luo S. Xiao X. Guo H. Chen J. Wan Y. . (2010). Application of plant growth-promoting endophytes (PGPE) isolated from *Solanum nigrum* L. for phytoextraction of cd-polluted soils. Appl. Soil Ecol. 46, 383–389. doi: 10.1016/j.apsoil.2010.10.003

[ref19] Chen Q. Zhao X. Lei D. Hu S. Shen Z. Shen W. . (2017). Hydrogen-rich water pretreatment alters photosynthetic gas exchange, chlorophyll fluorescence, and antioxidant activities in heat-stressed cucumber leaves. Plant Growth Regul. 83, 69–82. doi: 10.1007/s10725-017-0284-1

[ref20] Chen S. Zhou Y. Chen Y. Gu J. (2018). Fastp: an ultra-fast all-in-one FASTQ preprocessor. Bioinformatics 34, i884–i890. doi: 10.1093/bioinformatics/bty560, 30423086 PMC6129281

[ref21] Chi Y. Ma X. Zhang X. Wang R. Zhang D. Chu S. . (2024). Plant growth promoting endophyte modulates soil ecological characteristics during the enhancement process of cadmium phytoremediation. J. Environ. Manag. 369:122206. doi: 10.1016/j.jenvman.2024.122206, 39197342

[ref22] Choppala G. Kunhikrishnan A. Seshadri B. Park J. H. Bush R. Bolan N. (2018). Comparative sorption of chromium species as influenced by pH, surface charge and organic matter content in contaminated soils. J. Geochem. Explor. 184, 255–260. doi: 10.1016/j.gexplo.2016.07.012

[ref23] Conlon R. Dowling D. N. Germaine K. J. (2025). Assessing microbial activity and rhizoremediation in hydrocarbon and heavy metal-impacted soil. Microorganisms 13:848. doi: 10.3390/microorganisms13040848, 40284684 PMC12029208

[ref24] Deng D. M. Shu W. S. Zhang J. Zou H. L. Lin Z. Ye Z. H. . (2007). Zinc and cadmium accumulation and tolerance in populations of *Sedum alfredii*. Environ. Pollut. 147, 381–386. doi: 10.1016/j.envpol.2006.05.024, 16828210

[ref25] Doolotkeldieva T. Bobusheva S. Konurbaeva M. (2024). In vitro and in vivo screening of bacterial species from contaminated soil for heavy metal biotransformation activity. J. Environ. Sci. Health B 59, 315–332. doi: 10.1080/03601234.2024.2343236, 38676363

[ref26] Edgar R. C. (2013). UPARSE: highly accurate OTU sequences from microbial amplicon reads. Nat. Methods 10, 996–998. doi: 10.1038/nmeth.2604, 23955772

[ref27] Edwards J. Johnson C. Santos-Medellín C. Lurie E. Podishetty N. K. Bhatnagar S. . (2015). Structure, variation, and assembly of the root-associated microbiomes of rice. PNAS 112, E911–E920. doi: 10.1073/pnas.1414592112, 25605935 PMC4345613

[ref28] Elzinga E. J. Cirmo A. (2010). Application of sequential extractions and X-ray absorption spectroscopy to determine the speciation of chromium in northern New Jersey marsh soils developed in chromite ore processing residue (COPR). J. Hazard. Mater. 183, 145–154. doi: 10.1016/j.jhazmat.2010.06.130, 20674158

[ref29] Fan M. Liu Z. Nan L. Wang E. Chen W. Lin Y. . (2018). Isolation, characterization, and selection of heavy metal-resistant and plant growth-promoting endophytic bacteria from root nodules of *Robinia pseudoacacia* in a Pb/Zn mining area. Microbiol. Res. 217, 51–59. doi: 10.1016/j.micres.2018.09.002, 30384908

[ref30] Gao X. Li B. Yuan X. Yang Y. Lv M. Zhu Z. . (2025). Potential of pigeon pea [*Cajanus cajan* (L.) Millsp.] associated with endophytic bacterium *Bacillus cereus* PEB-9 to remediate cadmium-contaminated soil. J. Hazard. Mater. 493:138344. doi: 10.1016/j.jhazmat.2025.138344, 40267713

[ref31] Geesink P. Ter horst J. Ettema T. J. G. (2024). More than the sum of its parts: uncovering emerging effects of microbial interactions in complex communities. FEMS Microbiol. Ecol. 100:fiae029. doi: 10.1093/femsec/fiae029, 38444203 PMC10950044

[ref32] He C. Wang W. Hou J. (2020). Plant performance of enhancing licorice with dual inoculating dark septate endophytes and *Trichoderma viride* mediated via effects on root development. BMC Plant Biol. 20:325. doi: 10.1186/s12870-020-02535-9, 32646473 PMC7346674

[ref33] He X. Xiao W. Zeng J. Tang J. Wang L. (2023). Detoxification and removal of arsenite by *Pseudomonas* sp. SMS11: oxidation, biosorption and bioaccumulation. J. Environ. Manag. 336:117641. doi: 10.1016/j.jenvman.2023.117641, 36868151

[ref34] He X. Xu M. Wei Q. Tang M. Guan L. Lou L. . (2020). Promotion of growth and phytoextraction of cadmium and lead in *Solanum nigrum* L. mediated by plant-growth-promoting rhizobacteria. Ecotoxicol. Environ. Saf. 205:111333. doi: 10.1016/j.ecoenv.2020.111333, 32979802

[ref35] Herpell J. B. Alickovic A. Diallo B. Schindler F. Weckwerth W. (2023). Phyllosphere symbiont promotes plant growth through ACC deaminase production. ISME J. 17, 1267–1277. doi: 10.1038/s41396-023-01428-7, 37264153 PMC10356760

[ref36] Hou D. Lin Z. Wang R. Ge J. Wei S. Xie R. . (2018). Cadmium exposure-*Sedum alfredii* planting interactions shape the bacterial community in the hyperaccumulator plant rhizosphere. Appl. Environ. Microbiol. 84, e02797–e02717. doi: 10.1128/AEM.02797-17, 29654182 PMC5981072

[ref37] Hu L. Liu B. Li S. Zhong H. He Z. (2021). Study on the oxidative stress and transcriptional level in Cr(VI) and hg(II) reducing strain *Acinetobacter indicus* yy-1 isolated from chromium-contaminated soil. Chemosphere 269:128741. doi: 10.1016/j.chemosphere.2020.128741, 33127119

[ref38] Jan R. Khan M. A. Asaf S. Lubna Lee I.-J. Kim K. M. (2019). Metal resistant endophytic bacteria reduces cadmium, nickel toxicity, and enhances expression of metal stress related genes with improved growth of *Oryza Sativa*, via regulating its antioxidant machinery and endogenous hormones. Plants 8:363. doi: 10.3390/plants8100363 31547575 PMC6844085

[ref39] Jeyasundar P. G. S. A. Ali A. Azeem M. Li Y. Guo D. Sikdar A. . (2021). Green remediation of toxic metals contaminated mining soil using bacterial consortium and *Brassica juncea*. Environ. Pollut. 277:116789. doi: 10.1016/j.envpol.2021.116789, 33640810

[ref40] Jiang W. Wang S. Gu F. Yang X. Qi Q. Liang Q. (2025). Advances in synthetic microbial ecosystems approach for studying ecological interactions and their influencing factors. Eng. Microbiol. 5:100205. doi: 10.1016/j.engmic.2025.100205 PMC12967826 41982405

[ref41] Jiang R. Zhu C. Wen S. Zhang M. Hou X. (2025). Phosphate-solubilizing bacteria for lead heavy metal phytoremediation by reducing bermudagrass stress and enhancing lead bioaccumulation. Ecotoxicol. Environ. Saf. 299:118371. doi: 10.1016/j.ecoenv.2025.118371, 40398251

[ref42] Ju W. Liu L. Fang L. Cui Y. Duan C. Wu H. (2019). Impact of co-inoculation with plant-growth-promoting rhizobacteria and rhizobium on the biochemical responses of alfalfa-soil system in copper contaminated soil. Ecotoxicol. Environ. Saf. 167, 218–226. doi: 10.1016/j.ecoenv.2018.10.016, 30342354

[ref43] Khan A. R. Ullah I. Khan A. L. Park G.-S. Waqas M. Hong S.-J. . (2015). Improvement in phytoremediation potential of *Solanum nigrum* under cadmium contamination through endophytic-assisted *Serratia* sp. RSC-14 inoculation. Environ. Sci. Pollut. Res. Int. 22, 14032–14042. doi: 10.1007/s11356-015-4647-8, 25956518

[ref44] Kumar A. Tripti Voropaeva O. Maleva M. Panikovskaya K. Borisova G. . (2021). Bioaugmentation with copper tolerant endophyte *Pseudomonas lurida* strain EOO26 for improved plant growth and copper phytoremediation by *Helianthus annuus*. Chemosphere 266:128983. doi: 10.1016/j.chemosphere.2020.128983 33272662

[ref45] Li X. Q. Liu Y. Q. Li Y. J. Han H. Zhang H. Ji M. F. . (2022). Enhancing mechanisms of the plant growth-promoting bacterial strain *Brevibacillus* sp. SR-9 on cadmium enrichment in sweet sorghum by metagenomic and transcriptomic analysis. Int. J. Environ. Res. Public Health 19:16309. doi: 10.3390/ijerph192316309, 36498382 PMC9737414

[ref46] Li T. Xu Z. Han X. Yang X. Sparks D. L. (2012). Characterization of dissolved organic matter in the rhizosphere of hyperaccumulator *Sedum alfredii* and its effect on the mobility of zinc. Chemosphere 88, 570–576. doi: 10.1016/j.chemosphere.2012.03.031, 22475152

[ref47] Lichtenthaler H. K. (1987). “Chlorophylls and carotenoids: pigments of photosynthetic biomembranes” in Methods in Enzymology, Eds. S.P Colowick, N.O Kaplan vol. 148 (San Diego: Academic Press), 350–382.

[ref48] Lin Z. Qiao Y. Ge J. Lu L. Xie R. Tian S. (2025). Novel plant growth-promoting endophytic bacteria, *Stenotrophomonas maltophilia* SaRB5, facilitate phytoremediation by plant growth and cadmium absorption in *Salix suchowensis*. Ecotoxicol. Environ. Saf. 303:118967. doi: 10.1016/j.ecoenv.2025.118967, 40925184

[ref49] Liu H. Cheng H. Xu S. Zhang D. Wu J. Li Z. . (2025). Genetic diversity and growth-promoting functions of endophytic nitrogen-fixing bacteria in apple. Plants (Basel) 14:1235. doi: 10.3390/plants14081235, 40284123 PMC12030322

[ref50] Liu C. Lin H. Dong Y. Li B. (2022). Increase of P and cd bioavailability in the rhizosphere by endophytes promoted phytoremediation efficiency of *Phytolacca acinosa*. J. Hazard. Mater. 431:128546. doi: 10.1016/j.jhazmat.2022.128546, 35278959

[ref51] Liu L. Quan S. Li L. Lei G. Li S. Gong T. . (2024). Endophytic bacteria improve bio- and phytoremediation of heavy metals. Microorganisms 12:2137. doi: 10.3390/microorganisms12112137, 39597526 PMC11597072

[ref52] Long Y. Yu G. Wang J. Zheng D. (2024). Cadmium removal by constructed wetlands containing different substrates: performance, microorganisms and mechanisms. Bioresour. Technol. 413:131561. doi: 10.1016/j.biortech.2024.131561, 39362346

[ref53] Lyu Y. Yang T. Liu H. Qi Z. Li P. Shi Z. . (2021). Enrichment and characterization of an effective hexavalent chromium-reducing microbial community YEM001. Environ. Sci. Pollut. Res. Int. 28, 19866–19877. doi: 10.1007/s11356-020-11863-0, 33410044

[ref54] Ma Y. Prasad M. N. V. Rajkumar M. Freitas H. (2011). Plant growth promoting rhizobacteria and endophytes accelerate phytoremediation of metalliferous soils. Biotechnol. Adv. 29, 248–258. doi: 10.1016/j.biotechadv.2010.12.001, 21147211

[ref55] Ma Y. Rajkumar M. Zhang C. Freitas H. (2016). Beneficial role of bacterial endophytes in heavy metal phytoremediation. J. Environ. Manag. 174, 14–25. doi: 10.1016/j.jenvman.2016.02.047, 26989941

[ref56] Magoč T. Salzberg S. L. (2011). FLASH: fast length adjustment of short reads to improve genome assemblies. Bioinformatics 27, 2957–2963. doi: 10.1093/bioinformatics/btr507, 21903629 PMC3198573

[ref57] Malaviya P. Singh A. (2016). Bioremediation of chromium solutions and chromium containing wastewaters. Crit. Rev. Microbiol. 42, 607–633. doi: 10.3109/1040841x.2014.974501, 25358056

[ref58] Mishra S. Bharagava R. N. (2016). Toxic and genotoxic effects of hexavalent chromium in environment and its bioremediation strategies. J. Environ. Sci. Health C Environ. Carcinog. Ecotoxicol. Rev. 34, 1–32. doi: 10.1080/10590501.2015.1096883, 26398402

[ref59] Naziębło A. Dobrzyński J. (2025). Biotransformation of as, Cr, hg, and Mn by Pseudomonadota: chances and risks. Biodegradation 36:60. doi: 10.1007/s10532-025-10157-x, 40663258 PMC12263811

[ref60] Pacheco Da Silva M. L. Moen F. S. Liles M. R. Feng Y. Sanz-Saez A. (2022). The response to inoculation with PGPR plus orange peel amendment on soybean is cultivar and environment dependent. Plants 11:1138. doi: 10.3390/plants11091138, 35567141 PMC9104577

[ref61] Pan F. Meng Q. Luo S. Shen J. Chen B. Khan K. Y. . (2017). Enhanced cd extraction of oilseed rape (*Brassica napus*) by plant growth-promoting bacteria isolated from cd hyperaccumulator Sedum alfredii Hance. Int. J. Phytoremediation 19, 281–289. doi: 10.1080/15226514.2016.1225280, 27593491

[ref62] Pishchik V. N. Vorob’ev N. I. Provorov N. A. Khomyakov Y. V. (2016). Mechanisms of plant and microbial adaptation to heavy metals in plant-microbial systems. Microbiology 85, 257–271. doi: 10.1134/S0026261716030097

[ref63] Rao M. C. S. Rahul V. D. Uppar P. Madhuri M. L. Tripathy B. Vyas R. D. V. . (2025). Enhancing the phytoremediation of heavy metals by plant growth promoting rhizobacteria (PGPR) consortium: a narrative review. J. Basic Microbiol. 65:e2400529. doi: 10.1002/jobm.202400529, 39462911

[ref64] Ren H. Guo Y. Zhang S. Wang Y. Zhou J. Chen H. (2025). Endophytic bacteria enhance cadmium remediation through siderophore production and soil microbial dynamics. Environ. Technol. 46, 5649–5663. doi: 10.1080/09593330.2025.2550674, 40877206

[ref65] Saharan B. S. Chaudhary T. Mandal B. S. Kumar D. Kumar R. Sadh P. K. . (2023). Microbe-plant interactions targeting metal stress: new dimensions for bioremediation applications. J. Xenobiot. 13, 252–269. doi: 10.3390/jox13020019, 37367495 PMC10304886

[ref66] Sahoo R. Sow S. Ranjan S. Dharminder Kumar R. Roy D. K. . (2024). Unveiling the potential of plant growth promoting rhizobacteria (PGPR) in phytoremediation of heavy metal. Discov. Appl. Sci. 6:324. doi: 10.1007/s42452-024-06024-8

[ref67] Schue M. Fekete A. Ortet P. Brutesco C. Heulin T. Schmitt-Kopplin P. . (2011). Modulation of metabolism and switching to biofilm prevail over exopolysaccharide production in the response of *Rhizobium alamii* to cadmium. PLoS One 6:e26771. doi: 10.1371/journal.pone.0026771, 22096497 PMC3212527

[ref68] Schulte E. E. Hopkins B. G. (1996). “Estimation of soil organic matter by weight loss-on-ignition” in Soil organic matter: Analysis and interpretation. eds. Magdoff F. R. Tabatabai M. A. Hanlon E. A. Jr. (San Diego: The Soil Science Society of America, Inc.), 21–31.

[ref69] Shahzad R. Bilal S. Imran M. Khan A. L. Alosaimi A. A. Al-Shwyeh H. A. . (2019). Amelioration of heavy metal stress by endophytic *Bacillus amyloliquefaciens* RWL-1 in rice by regulating metabolic changes: potential for bacterial bioremediation. Biochem. J. 476, 3385–3400. doi: 10.1042/bcj20190606, 31696207

[ref70] Shi S. Nuccio E. Herman D. J. Rijkers R. Estera K. Li J. . (2015). Successional trajectories of rhizosphere bacterial communities over consecutive seasons. MBio 6:e00746. doi: 10.1128/mBio.00746-15, 26242625 PMC4526712

[ref71] Stackebrandt E. Goebel B. M. (1994). Taxonomic note: a place for DNA-DNA reassociation and 16S rRNA sequence analysis in the present species definition in bacteriology. Int. J. Syst. Evol. Microbiol. 44, 846–849. doi: 10.1099/00207713-44-4-846

[ref72] Świątczak J. Kalwasińska A. Brzezinska M. S. (2024). Plant growth–promoting rhizobacteria: *Peribacillus frigoritolerans* 2RO30 and *Pseudomonas sivasensis* 2RO45 for their effect on canola growth under controlled as well as natural conditions. Front. Plant Sci. 14:1233237. doi: 10.3389/fpls.2023.1233237, 38259930 PMC10800854

[ref73] Ullah I. Al-Johny B. O. Al-Ghamdi K. M. S. Al-Zahrani H. a. A. Anwar Y. Firoz A. . (2019). Endophytic bacteria isolated from *Solanum nigrum* L., alleviate cadmium (cd) stress response by their antioxidant potentials, including SOD synthesis by *sodA* gene. Ecotoxicol. Environ. Saf. 174, 197–207. doi: 10.1016/j.ecoenv.2019.02.074, 30826546

[ref74] Wang Q. Garrity G. M. Tiedje J. M. Cole J. R. (2007). Naïve Bayesian classifier for rapid assignment of rRNA sequences into the new bacterial taxonomy. Appl. Environ. Microbiol. 73, 5261–5267. doi: 10.1128/AEM.00062-07, 17586664 PMC1950982

[ref75] Wang Q. Ge C. Xu S. A. Wu Y. Sahito Z. A. Ma L. . (2020). The endophytic bacterium Sphingomonas SaMR12 alleviates cd stress in oilseed rape through regulation of the GSH-AsA cycle and antioxidative enzymes. BMC Plant Biol. 20:63. doi: 10.1186/s12870-020-2273-1, 32028891 PMC7006384

[ref76] Wang L. Lin H. Dong Y. Li B. He Y. (2020). Effects of endophytes inoculation on rhizosphere and endosphere microecology of Indian mustard (*Brassica juncea*) grown in vanadium-contaminated soil and its enhancement on phytoremediation. Chemosphere 240:124891. doi: 10.1016/j.chemosphere.2019.124891, 31574442

[ref77] Wang X. Luo S. Chen Y. Zhang R. Lei L. Lin K. . (2023). Potential of *Miscanthus floridulus* associated with endophytic bacterium *Bacillus cereus* BL4 to remediate cadmium contaminated soil. Sci. Total Environ. 857:159384. doi: 10.1016/j.scitotenv.2022.159384, 36240921

[ref78] Wang Q. Ma L. Zhou Q. Chen B. Zhang X. Wu Y. . (2019). Inoculation of plant growth promoting bacteria from hyperaccumulator facilitated non-host root development and provided promising agents for elevated phytoremediation efficiency. Chemosphere 234, 769–776. doi: 10.1016/j.chemosphere.2019.06.132, 31238273

[ref79] Wang Q. Zhou Q. Huang L. Xu S. Fu Y. Hou D. . (2022). Cadmium phytoextraction through *Brassica juncea* L. under different consortia of plant growth-promoting bacteria from different ecological niches. Ecotoxicol. Environ. Saf. 237:113541. doi: 10.1016/j.ecoenv.2022.113541, 35483144

[ref80] Wu K. Luo J. Li J. An Q. Yang X. Liang Y. . (2018). Endophytic bacterium *Buttiauxella* sp. SaSR13 improves plant growth and cadmium accumulation of hyperaccumulator *Sedum alfredii*. Environ. Sci. Pollut. Res. Int. 25, 21844–21854. doi: 10.1007/s11356-018-2322-6, 29796886

[ref81] Xiang J. Li N. Feng J. Yin J. Wang Y. Wang H. . (2024). Endophytic consortium exhibits varying effects in mitigating cadmium toxicity in rice cultivars with distinct cadmium accumulation capacities. Environ. Technol. Innov. 36:103833. doi: 10.1016/j.eti.2024.103833

[ref82] Xu J. Y. Han Y. H. Chen Y. Zhu L. J. Ma L. Q. (2016). Arsenic transformation and plant growth promotion characteristics of as-resistant endophytic bacteria from as-hyperaccumulator *Pteris vittata*. Chemosphere 144, 1233–1240. doi: 10.1016/j.chemosphere.2015.09.102, 26469935

[ref83] Yao Y. Hu L. Li S. Zeng Q. Zhong H. He Z. (2020). Exploration on the bioreduction mechanisms of Cr(VI) and hg(II) by a newly isolated bacterial strain *Pseudomonas umsongensis* CY-1. Ecotoxicol. Environ. Saf. 201:110850. doi: 10.1016/j.ecoenv.2020.110850, 32531571

[ref84] Yue Z. Chen Y. Chen C. Ma K. Tian E. Wang Y. . (2021). Endophytic *Bacillus altitudinis* WR10 alleviates cu toxicity in wheat by augmenting reactive oxygen species scavenging and phenylpropanoid biosynthesis. J. Hazard. Mater. 405:124272. doi: 10.1016/j.jhazmat.2020.124272, 33097348

[ref85] Yung L. Blaudez D. Maurice N. Azou-Barré A. Sirguey C. (2021). Dark septate endophytes isolated from non-hyperaccumulator plants can increase phytoextraction of cd and Zn by the hyperaccumulator *Noccaea caerulescens*. Environ. Sci. Pollut. Res. Int. 28, 16544–16557. doi: 10.1007/s11356-020-11793-x, 33387325

[ref86] Zhang J. Wang L. H. Yang J. C. Liu H. Dai J. L. (2015). Health risk to residents and stimulation to inherent bacteria of various heavy metals in soil. Sci. Total Environ. 508, 29–36. doi: 10.1016/j.scitotenv.2014.11.064, 25437950

[ref87] Zheng X. Tong J. Zhou S. Liu Y. Liu G. Zou D. (2024). Remediation of hexavalent chromium contaminated soils by stimulating indigenous microorganisms: optimization, community succession and applicability. J. Environ. Manag. 372:123222. doi: 10.1016/j.jenvman.2024.123222, 39549449

